# circDHTKD1 promotes lymphatic metastasis of bladder cancer by upregulating CXCL5

**DOI:** 10.1038/s41420-022-01037-x

**Published:** 2022-05-03

**Authors:** Qun Lu, Haoli Yin, Yongming Deng, Wei Chen, Wenli Diao, Meng Ding, Wenmin Cao, Yao Fu, Wenjing Mo, Xiaoqing Chen, Qing Zhang, Xiaozhi Zhao, Hongqian Guo

**Affiliations:** 1 Department of Urology, Affiliated Drum Tower Hospital, Medical School of Nanjing University, Institute of Urology, Nanjing University, Nanjing, Jiangsu China; 2 Department of Pathology, Affiliated Drum Tower Hospital, Medical School of Nanjing University, Nanjing, Jiangsu China

**Keywords:** Non-coding RNAs, Bladder cancer

## Abstract

Lymph node (LN) metastasis is associated with unfavorable prognosis of bladder cancer (BCa). Although lymphangiogenesis is functionally important in LN metastasis of tumors, the potential mechanism in BCa remains unclear. Here, we clarified a regulatory mechanism of circRNA-mediated lymphangiogenesis and LN metastasis in BCa based on next-generation sequencing data. We revealed that circDHTKD1 was positively associated with LN metastasis and significantly upregulated in BCa. By analyzing the co-expression patterns of circDHTKD1 and differentially expressed mRNAs, we identified that circDHTKD1 facilitated lymphangiogenesis by upregulating CXCL5. Mechanistically, circDHTKD1 directly interacted with miR-149-5p, and antagonized the repression of miR-149-5p on CXCL5. Furthermore, circDHTKD1-induced CXCL5 expression recruited and activated neutrophils, which participated in lymphangiogenesis by secreting VEGF-C. Our study supports circDHTKD1 as a promising diagnostic and therapeutic target for LN metastasis in BCa.

## Introduction

Bladder cancer (BCa) is a prevalent urologic malignant tumor with a high rate of mortality and morbidity [1]. Approximately 20-30% of patients have muscle-invasive BCa [2], which is associated with frequent metastasis and poor prognosis [3]. Despite considerable progress in therapeutic strategies for BCa, the overall survival rate remains at a low level [4]. Lymph node (LN) metastasis is associated with unfavorable prognosis of BCa, which reduces the 5-year survival rate to only 18.6% [5]. In LN metastasis disease, the lymphatic vessels undergo dynamic changes including lymphangiogenesis, which means new lymphatic vessels forming from existing ones [6]. Although lymphangiogenesis is functionally important in LN metastasis of tumors [7, 8], the potential mechanism in BCa remains unclear. Therefore, it is important to explore the mechanisms regulating LN metastasis of BCa to develop more precise therapeutic strategies.

Circular RNAs (circRNAs) are a group of noncoding molecules, that display covalently closed loops with neither 5′ cap nor 3′poly A tail [9]. CircRNAs have many specific characteristics, including stability, high abundance, conservation, and tissue specificity [10, 11]. With the development of sequencing technology, large numbers of circRNAs have been detected, indicating multiple potential regulatory roles in biological processes [12]. Many studies have shown that circRNAs play important roles in BCa progression [13–15], and can function as ‘microRNA (miRNA) sponges’ for containing miRNA binding elements [16]. CircRNAs competitively combine with miRNAs to affect the translation of downstream mRNA and signaling pathway transduction [17]. Nevertheless, little is known about the roles of circRNAs in LN metastasis of BCa, and the regulatory mechanisms need further exploration [18].

Here, we found that circDHTKD1 (hsa_circ_0007813) was positively associated with LN metastasis and significantly upregulated in BCa. Mechanistically, circDHTKD1 promoted lymphangiogenesis in BCa by sponging miR-149-5p to upregulate CXCL5. Furthermore, circDHTKD1-induced CXCL5 expression in BCa recruited neutrophils, which participated in lymphangiogenesis by secreting VEGF-C. These findings reveal that circDHTKD1 plays an oncogenic role and may be a candidate for the diagnosis and therapy of LN metastasis in BCa.

## Results

### circDHTKD1 (hsa_circ_0007813) correlates with LN metastasis of BCa

**Fig. 1:**
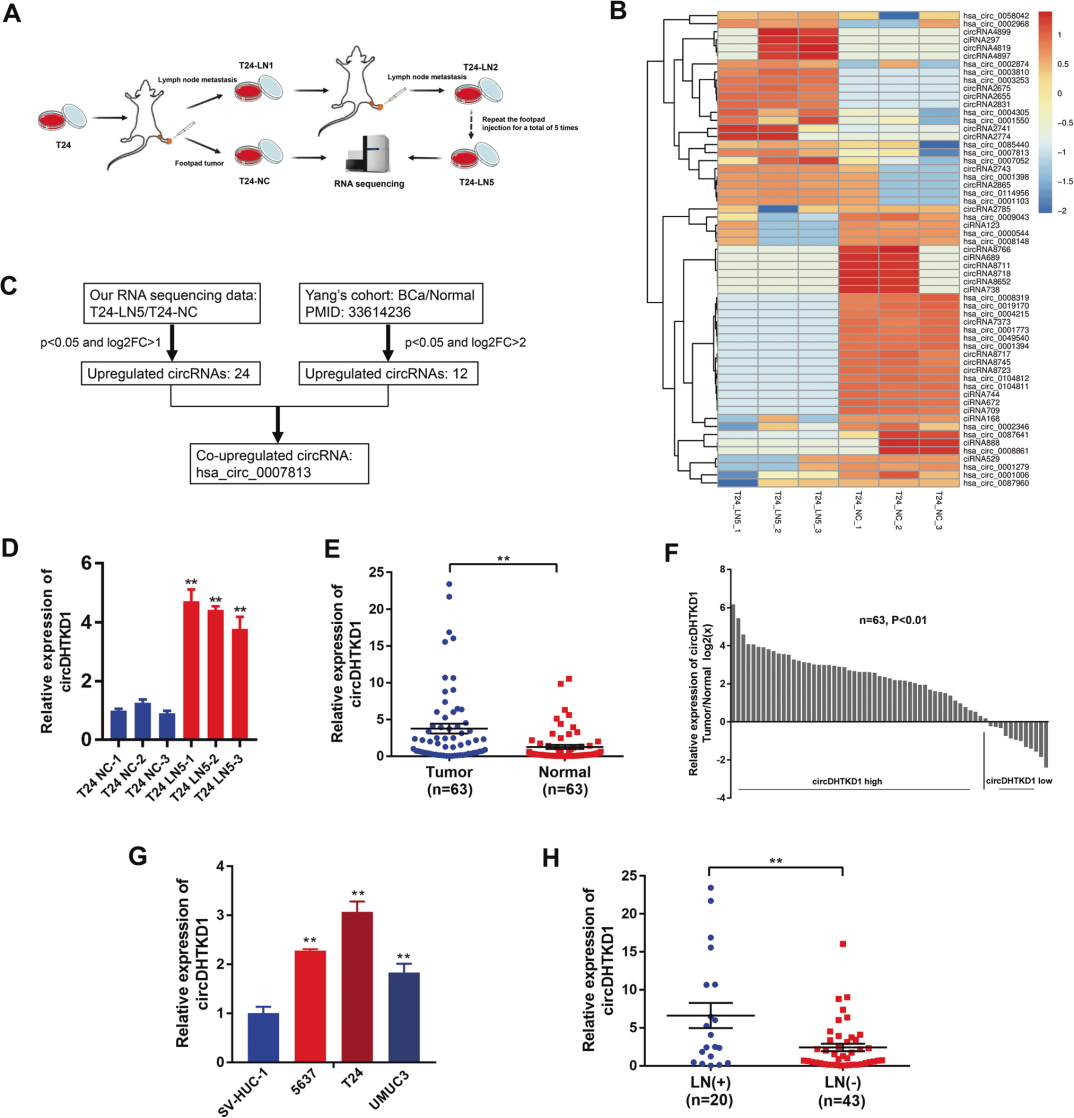
circDHTKD1 (hsa_circ_0007813) correlates with LN metastasis of BCa. **A** The overall scheme of popliteal LN metastasis model establishment to achieve T24 cells with high LN metastatic capacity (T24-LN5). **B** Heatmap of upregulated circRNAs in T24-LN5 cells compared to T24-NC cells. **C** Schematic diagram of circDHTKD1 upregulation in T24-LN5 and BCa tissues. **D** The level of circDHTKD1 was validated by qRT-PCR in 3 pairs of T24-LN5/T24-NC cells. **E** and **F** qRT-PCR confirmed the circDHTKD1 expression in 63 pairs of BCa tissues and matched normal tissues. **G** qRT-PCR detected circDHTKD1 expression in SV-HUC-1 and BCa cells. **H** Analysis of circDHTKD1 expression in 63 BCa tissues according to LN status. Error bars indicate standard deviations of three independent experiments. *p < 0.05 and **p < 0.01.

We used the popliteal LN metastasis model, which simulated the lymphatic drainage and metastasis of BCa, to achieve T24 cells with high LN-metastatic capacity (Fig. 1A). T24 cells were inoculated into the footpads of each mouse. When popliteal LN metastasis occurred, the footpad tumor and enlarged popliteal LNs were excised and cut into small pieces for culture. The tumor cells from the footpad tumor were considered as control (T24-NC). The tumor cells from the popliteal LNs (T24-LN1) were injected repeatedly into the footpad 4 more times following the above steps to achieve T24 cells with high LN-metastatic capacity (T24-LN5). T24-LN5/T24-NC cells were inoculated into the footpads of each mouse, and the volume of popliteal LNs was larger in the T24-LN5 group (Fig. S1A). To identify critical circRNAs that promote LN metastasis, next-generation sequencing was used to analyze three pairs of T24-LN5/T24-NC cells (Fig. 1B). Besides, we studied published sequencing data concerning the circRNA profiles in BCa tissues [19], and found that circDHTKD1 (hsa_circ_0007813) was consistently upregulated in the two sets of data (Fig. 1C). The expression of circDHTKD1 was validated in the sequenced cell samples and 63 pairs of BCa tissues and paired normal tissues. We found that circDHTKD1 was dramatically overexpressed in T24-LN5 cells and BCa tissues (Fig. 1D-F). Additionally, upregulation of circDHTKD1 was also found in 5637, T24 and UMUC3 cells, compared with normal urothelial cell line SV-HUC-1 (Fig. 1G). Analysis of the BCa tissues revealed that circDHTKD1 expression was positively correlated with LN metastasis of BCa (Fig. 1H).

### Characteristics of circDHTKD1 in BCa cells

**Fig. 2:**
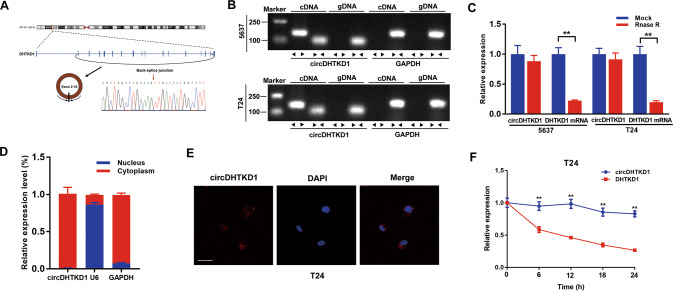
Characteristics of circDHTKD1 in BCa cells. **A** Schematic diagram showing the circularization of circDHTKD1 derived from exons 2 to 16 of *DHTKD1*. The PCR product of circDHTKD1 was verified by Sanger sequencing. Red arrow indicates the back-splice junction site of circDHTKD1. **B** PCR for circDHTKD1 and DHTKD1 in cDNA and gDNA of 5637 and T24. GAPDH was applied as negative control. **C** qRT-PCR was used to detect the expression of circDHTKD1 and DHTKD1 mRNA in 5637 and T24 after treatment with RNase R. **D** and **E** RNA fractionation and FISH assay in T24 cells to detect the location of circDHTKD1. Scale bars, 20 µm. **F** qRT-PCR was used to detect the abundance of circDHTKD1 and DHTKD1 mRNA in T24 cells after treatment with Actinomycin D. Error bars indicate standard deviations of three independent experiments. *p < 0.05 and **p < 0.01.

circDHTKD1 arose from exons 2 to 16 of the *DHTKD1* gene (2504 bp). Next, the head-to-tail splicing of circDHTKD1 was confirmed by Sanger sequencing (Fig. 2A). Subsequently, DHTKD1 mRNA and circDHTKD1 were amplified by convergent primers and divergent primers. CircDHTKD1 could only be amplified in cDNA, not in genomic DNA (gDNA) (Fig. 2B). The stability of circDHTKD1 was further studied. After RNase R treatment, circDHTKD1 was retained, while linear DHTKD1 mRNA expression decreased dramatically (Fig. 2C). Further examination by RNA fractionation and FISH in T24 cells revealed that circDHTKD1 was primarily localized in the cytoplasm (Fig. 2D-E). We also analyzed the stability of circDHTKD1 and DHTKD1 mRNA treated with Actinomycin D, and revealed a longer half-life of circDHTKD1 than DHTKD1 mRNA (Fig. 2F). These results confirm the characteristics of circDHTKD1 as a circRNA.

### circDHTKD1 promotes the progression of BCa cells

**Fig. 3:**
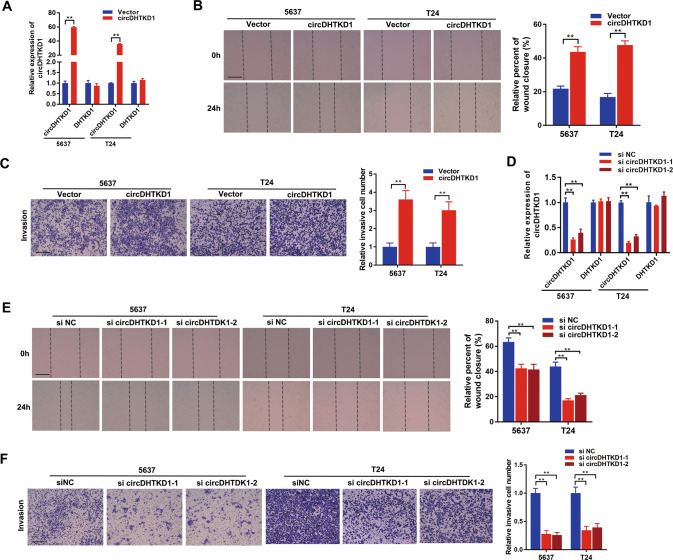
circDHTKD1 promotes the migration and invasion of BCa cells. **A** qRT-PCR was used to assess the expression of circDHTKD1 and DHTKD1 mRNA in BCa cells after transfection of circDHTKD1 expression vector or control vector. **B** Wound healing assay showing the migration capability after circDHTKD1 overexpression in BCa cells. **C** Transwell assay showing the invasion capability after circDHTKD1 overexpression in BCa cells. **D** qRT-PCR was used to assess the expression of circDHTKD1 and DHTKD1 mRNA after circDHTKD1 knockdown. **E** Wound healing assay showing the migration capability after circDHTKD1 knockdown in BCa cells. **F** Transwell assay showing the invasion capability after circDHTKD1 knockdown in BCa cells. Scale bars, 100 µm. Error bars indicate standard deviations of three independent experiments. *p < 0.05 and **p < 0.01.

LN metastasis is a complex and multifactorial process. The progression of the tumor itself are important factors in metastasis. The expression of circDHTKD1 was overexpressed by transfection with circDHTKD1 plasmids, and no apparent change was detected with DHTKD1 mRNA (Fig. 3A). The migration and invasion of BCa cells were significantly induced (Fig. 3B-C). Similarly, circDHTKD1 was knocked down by siRNAs targeting the junction site of circDHTKD1 without affecting DHTKD1 mRNA (Fig. 3D). Functional assays revealed that circDHTKD1 downregulation suppressed the migration and invasion abilities (Fig. 3E–F). Besides, CCK-8 and colony formation assays showed that circDHTKD1 overexpression promoted the proliferation ability, and circDHTKD1 knockdown inhibited the proliferation ability of BCa cells (Fig. S2A–H). The above findings reveal that circDHTKD1 promotes the migration, invasion, and proliferation of BCa cells.

### circDHTKD1 promotes lymphangiogenesis and LN metastasis of BCa

**Fig. 4:**
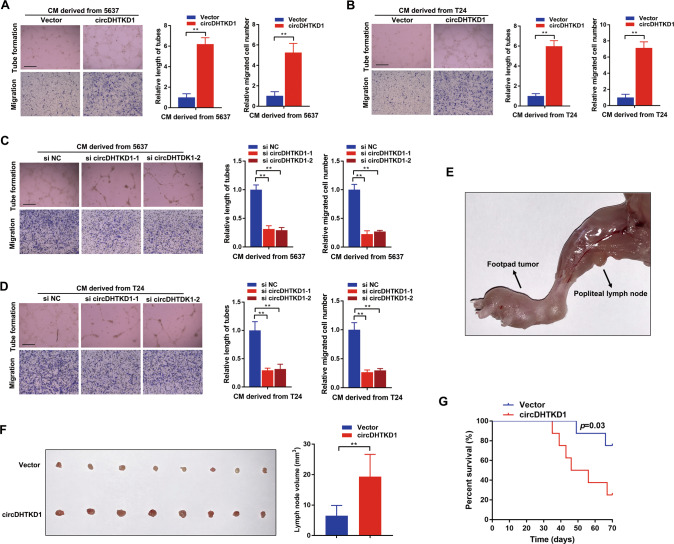
circDHTKD1 promotes lymphangiogenesis and LN metastasis of BCa. **A** and **B** Tube formation and Transwell migration of HLEC cultured with conditioned medium derived from circDHTKD1-upregulated or control BCa cells. **C** and **D** Tube formation and Transwell migration of HLEC cultured with conditioned medium from circDHTKD1-downregulated or control BCa cells. Scale bars, 100 µm. **E** An image of the popliteal LN metastasis model. Black arrow indicated the footpad tumor and popliteal LN. **F** Representative images of excised popliteal LNs and the measurement of the LN volume. **G** Kaplan–Meier analysis evaluating the survival. Error bars indicate standard deviations of three independent experiments. *p < 0.05 and **p < 0.01.

Lymphangiogenesis is considered a key factor for LN metastasis. The function of circDHTKD1 in LN metastasis was investigated. In vitro assays revealed that conditioned medium from circDHTKD1-overexpressing BCa cells significantly facilitated tube formation and migration of HLEC (Fig. 4A-B), while circDHTKD1 knockdown abolished this ability (Fig. 4C–D). A popliteal LN metastasis model was employed to detect the function of circDHTKD1 in LN metastasis (Fig. 4E). T24 cell lines stably overexpressing circDHTKD1 were inoculated into the footpads of each mouse. The status of metastasis was checked when the footpad tumors reached approximately 200 mm^3^. Strikingly, circDHTKD1 overexpression facilitated BCa metastasis to LNs, and the volume of popliteal LNs was larger (Fig. 4F). In addition, the survival was worse in the circDHTKD1-overexpressing group (Fig. 4G). We also found that the primary footpad tumor size in circDHTKD1-overexpressing group was larger than the control group at the same time point, suggesting that circDHTKD1 promoted the tumorigenesis of BCa (Fig. S2I). Collectively, these results suggest that circDHTKD1 facilitates lymphangiogenesis and LN metastasis of BCa.

### circDHTKD1 facilitates LN metastasis by targeting CXCL5 and sponges miR-149-5p in BCa

**Fig. 5:**
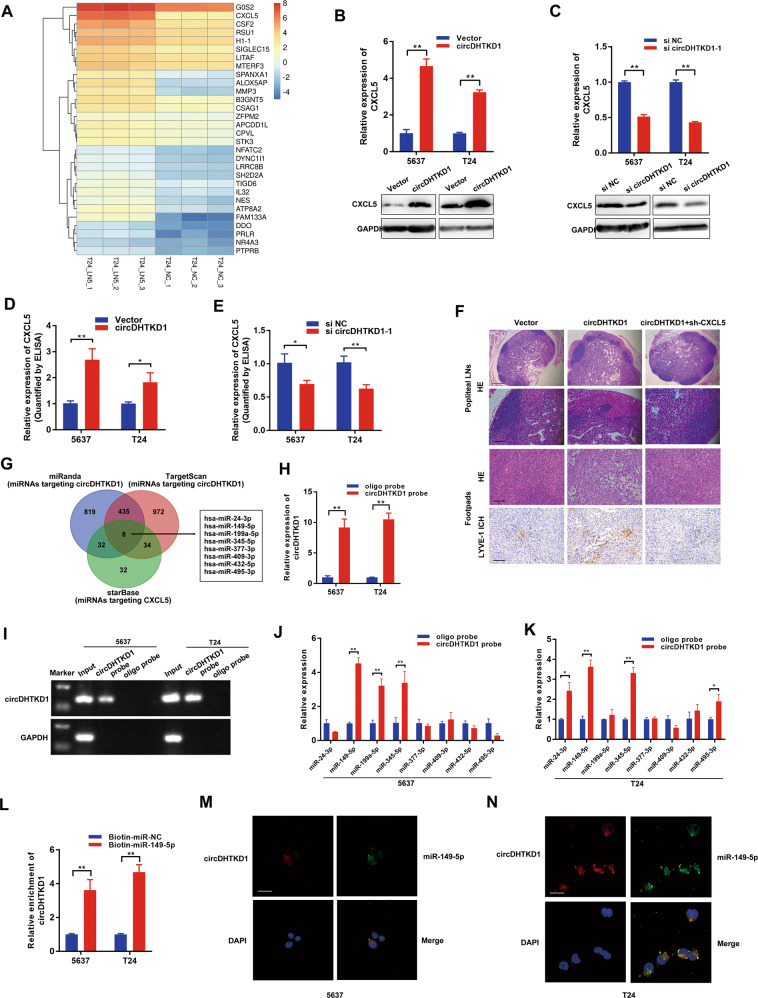
circDHTKD1 facilitates LN metastasis by targeting CXCL5 and sponges miR-149-5p in BCa. **A** A heatmap showing mRNA levels of 30 genes co-expressed with circDHTKD1 based on sequencing analysis. **B** and **C** The mRNA and protein expression of CXCL5 in BCa cells with overexpression or knockdown of circDHTKD1. **D** and **E** ELISA of CXCL5 in BCa cells with overexpression or knockdown of circDHTKD1. **F** Images of HE and IHC staining showing LN metastasis and lymphatic vessel quantities in footpad tumors in the indicated mouse group. Scale bars: red, 200 µm; black, 50 µm. **G** Schematic diagram showing overlapping of binding miRNAs of circDHTKD1 and CXCL5 predicted by miRanda, TargetScan, and starBase. **H** and **I** The enrichment of circDHTKD1 in BCa cell lysates with circDHTKD1 probe was detected by qRT-PCR. Relative level of circDHTKD1 was normalized to input. **J** and **K** Relative levels of 8 miRNA candidates in circDHTKD1 pull-down complexes were detected by qRT-PCR. **L** qRT-PCR analysis of circDHTKD1 captured by biotinylated miR-149-5p. **M** and **N** FISH showing the location of circDHTKD1 and miR-149-5p in BCa cells. Scale bars, 20 µm. Error bars indicate standard deviations of three independent experiments. *p < 0.05 and **p < 0.01.

We analyzed the co-expression patterns of circDHTKD1 and differentially expressed mRNAs based on their expressions in sequencing data, by calculating the Pearson correlation coefficient (PCC). In the analysis, we selected 30 putative co-expressed mRNAs based on the rank of PCC values (Fig. 5A). Among these, ALOX5AP, FAM133A, and CXCL5 were significantly upregulated in T24-LN5 cells (Fig. S3A). We further detected whether these three genes could be regulated by circDHTKD1, and found that circDHTKD1 expression was more positively correlated with CXCL5 than ALOX5AP and FAM133A expression (Fig. S3B-C). circDHTKD1 overexpression significantly induced the levels of CXCL5 mRNA and protein, and circDHTKD1 knockdown showed the opposite effects (Fig. 5B-C). The results were further confirmed by ELISA (Fig. 5D-E). We also found that CXCL5 was overexpressed in BCa tissues and positively correlated with LN metastasis (Fig. S4A-B). IHC analysis revealed that circDHTKD1 overexpression significantly increased LN metastasis and lymphatic vessel quantities in primary tumor tissues, which were decreased by CXCL5 knockdown (Fig. 5F). In vitro, CXCL5 knockdown inhibited circDHTKD1-induced tube formation and migration of HLEC (Fig. S4C). Immunofluorescence demonstrated the expression of CXCR2 in HLEC, which was the receptor of CXCL5 (Fig. S4D).

It is recognized that cytoplasm-located circRNAs can act as miRNA sponges to regulate downstream genes [20]. Therefore, 8 candidate miRNAs that contained common binding sites for circDHTKD1 and CXCL5 were predicted by miRanda, TargetScan, and starBase (Fig. 5G). Next, the interaction was confirmed by RNA pull-down assay. The biotin-labeled circDHTKD1 probe could enrich circDHTKD1 (Fig. 5H-I), and miR-149-5p was dramatically pulled down in both 5637 and T24 cells (Fig. 5J-K). Subsequently, biotin-labeled miR-149-5p mimics were applied to further verify the binding effect. We found that biotin-labeled miR-149-5p enriched more circDHTKD1 than the control (Fig. 5L). Moreover, FISH analysis revealed that circDHTKD1 and miR-149-5p were co-localized in the cytoplasm (Fig. 5M-N). Together, the results indicate that circDHTKD1 facilitates LN metastasis by targeting CXCL5 and sponges miR-149-5p in BCa.

### circDHTKD1 antagonizes the repression of miR-149-5p on CXCL5

**Fig. 6:**
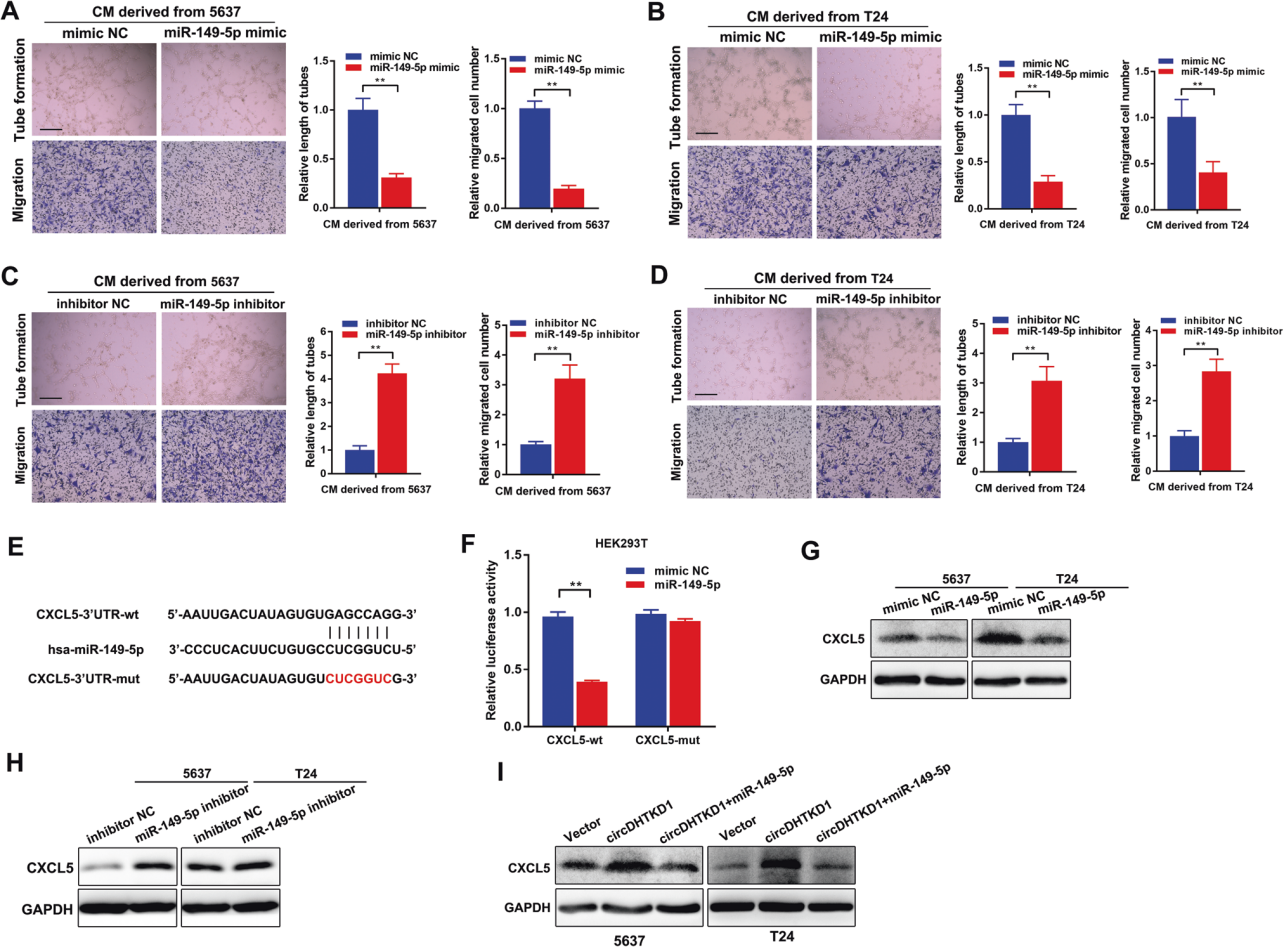
circDHTKD1 antagonizes the repression of miR-149-5p on CXCL5. **A** and **B** Tube formation and Transwell migration of HLEC cultured with conditioned medium from miR-149-5p-upregulated or control BCa cells. **C** and **D** Tube formation and Transwell migration of HLEC cultured with conditioned medium from miR-149-5p-downregulated or control BCa cells. Scale bars, 100 µm. **E** Predictive binding sequence of miR-149-5p in the 3’UTR of CXCL5. **F** Luciferase activities of CXCL5-3’UTR co-transfected with miR-149-5p or control mimics. **G** and **H** Western blot showing the CXCL5 levels after miR-149-5p overexpression and knockdown in BCa cells. **I** Western blot showing the effect of miR-149-5p upregulation on circDHTKD1-induced CXCL5 expression in BCa cells. Error bars indicate standard deviations of three independent experiments. *p < 0.05 and **p < 0.01.

Next, we assessed whether miR-149-5p mediated lymphangiogenesis in BCa. miR-149-5p expression was markedly upregulated by miR-149-5p mimics and downregulated by miR-149-5p inhibitors (Fig. S5A-B). miR-149-5p overexpression inhibited BCa cell-induced tube formation and migration of HLEC, while miR-149-5p silencing enhanced this effect (Fig. 6A-D). According to previous starBase prediction, miR-149-5p might bind to the 3′-UTR region of CXCL5 (Fig. 6E). We found that the luciferase activity of CXCL5-wt was strongly reduced by miR-149-5p, and mutated plasmids showed no obvious change in luciferase activity (Fig. 6F). Western blot demonstrated that miR-149-5p mimics could suppress the CXCL5 level, and miR-149-5p inhibitors could induce the CXCL5 level (Fig. 6G-H). Next, we investigated whether circDHTKD1 regulated the CXCL5 level by miR-149-5p. Western blot revealed that circDHTKD1 overexpression increased CXCL5 in BCa cells, and upregulation of miR-149-5p dramatically reversed this effect (Fig. 6I). Collectively, circDHTKD1 antagonizes the repression of miR-149-5p on the CXCL5 level.

### CXCL5-activated neutrophils induce lymphangiogenesis

**Fig. 7:**
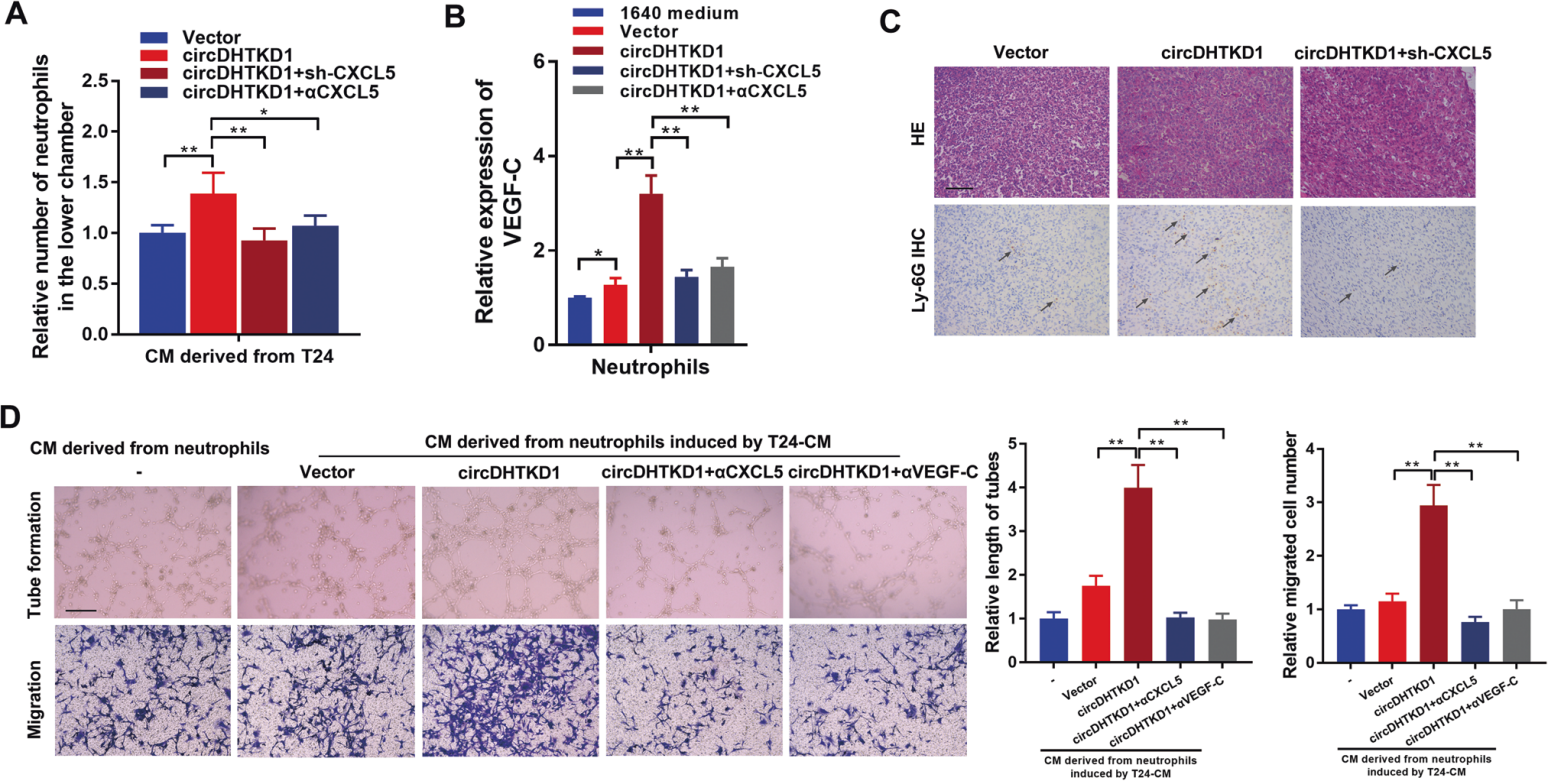
CXCL5-activated neutrophils induce lymphangiogenesis. **A** Chemotaxis of neutrophils induced by conditioned medium from BCa cells. **B** VEGF-C expression in neutrophils induced by conditioned medium was detected by qRT-PCR. **C** Neutrophils in footpad tumors in the indicated mouse group were evaluated by HE and IHC staining. Black arrows indicate the infiltrated neutrophils in footpad tumors. Scale bars, 50 µm. **D** Tube formation and Transwell migration of HLEC cultured with conditioned medium from neutrophils induced by indicated BCa cells. Scale bars, 100 µm. Error bars indicate standard deviations of three independent experiments. *p < 0.05 and **p < 0.01.

CXCL5 was a powerful neutrophil attractant [21]. Therefore, we examined whether CXCL5 acted on neutrophils and significantly influenced LN metastasis in BCa. We found that conditioned medium from circDHTKD1-overexpressing BCa cells induced the chemotaxis and VEGF-C expression of neutrophils compared with the control vector group, and CXCL5 knockdown or CXCL5-neutralizing antibody significantly suppressed this effect (Fig. 7A-B). Importantly, IHC revealed that circDHTKD1 overexpression induced neutrophil recruitment, which was suppressed by CXCL5 knockdown, indicating that neutrophils activated by circDHTKD1-upregulated CXCL5 participated in lymphangiogenesis (Fig. 7C). Moreover, adding CXCL5-neutralizing antibody to the medium of circDHTKD1-overexpressing BCa cells or adding VEGF-C-neutralizing antibody to the medium of neutrophils dramatically inhibited HLEC tube formation and migration (Fig. 7D). The results indicate that neutrophils activated by circDHTKD1-upregulated CXCL5 contribute to lymphangiogenesis by secreting VEGF-C.

## Discussion

LN metastasis is an indicator of poor prognosis for BCa patients. It is recognized that lymphangiogenesis is essential for cancer LN metastasis. Although VEGF-C plays a major role in lymphangiogenesis, studies have shown that a subset of patients with LN metastasis have low levels of VEGF-C [22]. In addition to growth factors of the VEGF family, many other mediators have been implicated in promoting lymphangiogenesis in cancer, including PDGF-BB, FGF2, and HGF [23–25]. Thus far, the exact mechanisms remain largely unknown. It is urgent to study the mechanisms of LN metastasis and find promising targets for treatment. Here, we found a circRNA, circDHTKD1, that promoted LN metastasis of BCa. Mechanistically, circDHTKD1 upregulated CXCL5 expression by sponging miR-149-5p, which promoted lymphangiogenesis in BCa. Furthermore, circDHTKD1-induced CXCL5 expression recruited and activated neutrophils, which induced lymphangiogenesis through VEGF-C upregulation. circDHTKD1 may be a novel intervention target for LN metastasis in BCa.

The expression of CXCL5 is upregulated in multiple malignant tumor types [26–28]. It was reported that CXCL5 could induce angiogenesis of colorectal cancer via upregulating FOXD1 [29]. The level of CXCL5 in LN metastasis was significantly higher than that in primary head and neck squamous cell carcinoma [30]. CXCL5 also plays a key role in lymphangiogenesis in melanoma, which can be inhibited by blocking the CXCR2 receptor [21]. At present, the specific regulatory mechanism of CXCL5 in LN metastasis of BCa remains unknown. We demonstrated that circDHTKD1-induced CXCL5 secretion promoted lymphangiogenesis in BCa, whereas CXCL5 knockdown significantly inhibited the above effect. We also demonstrated the expression of CXCR2 in HLEC, which was the receptor of CXCL5. The findings in this study clarified a regulatory mechanism of circRNA-mediated lymphangiogenesis and LN metastasis in BCa.

We also found that CXCL5 recruited neutrophils and participated in lymphangiogenesis by producing VEGF-C. Neutrophils can be recruited by CXCL5, which participates in tumor progression in the tumor microenvironment [31, 32]. CXCL5 promoted gastric cancer metastasis by activating neutrophils [33]. In melanoma, recruited neutrophils migrated through lymphatic vessels and promoted lymphatic metastasis [21]. However, there are few reports on how neutrophils promote LN metastasis, and the specific mechanism remains unclear. Our results revealed that circDHTKD1-induced CXCL5 recruited and activated neutrophils, which secreted higher level of VEGF-C that promoted lymphangiogenesis.

circRNAs are promising targets for diagnosis and treatment because of the features of high stability, unique structure, and specific expression pattern [34, 35]. However, the functions of circRNAs in LN metastasis of BCa are not clear. circEHBP1 promoted LN metastasis through activating the miR-130a-3p/VEGF-D pathway in BCa [18]. Our results revealed that circDHTKD1 was positively associated with clinical LN metastasis of BCa. Besides, circDHTKD1 upregulation significantly facilitated popliteal LN metastasis in vivo.

It has been confirmed that circRNAs harboring miRNA-binding elements could serve as miRNA sponges [16]. These circRNAs are mostly formed by the circularization of exons of protein-coding genes [12], and the subcellular location is mainly in the cytoplasm [20]. In the present study, we found that circDHTKD1 was associated with lymphatic metastasis and upregulated in BCa. circDHTKD1 was derived from the *DHTKD1* gene and located in the cytoplasm. Bioinformatic analyses were performed to screen miRNAs, that shared common binding sites with circDHTKD1 and CXCL5. We demonstrated that miR-149-5p could bind with circDHTKD1 and CXCL5, and circDHTKD1 significantly antagonized the repression of miR-149-5p on the CXCL5 level.

## Conclusions

In conclusion, our study demonstrated a novel mechanism by which circDHTKD1 increased CXCL5 expression by sponging miR-149-5p to accelerate LN metastasis in BCa. Furthermore, circDHTKD1-induced CXCL5 expression recruited and activated neutrophils, which participated in lymphangiogenesis. Our study supports circDHTKD1 as a promising diagnostic and therapeutic target for LN metastasis in BCa.

## Materials and methods

### Cell lines and human tissues

Bladder cancer cell lines 5637, T24, UMUC3, and normal urothelial epithelial cell line SV-HUC-1 were obtained from the Cell Bank of Type Culture Collection at Chinese Academy of Sciences. 5637 and T24 cells were cultured in 1640 medium, UMUC3 cells were cultured in DMEM, and SV-HUC-1 cells were cultured in F-12K medium. Human lymphatic endothelial cells (HLECs) were obtained from ScienCell Research Laboratories and cultured in ECM (ScienCell Research Laboratories, CA, USA). BCa tissues and matched normal tissues were obtained from Affiliated Drum Tower Hospital, Medical School of Nanjing University (Nanjing, China). The tissues were quickly stored in liquid nitrogen or −80 °C. All patients provided informed consent forms, and the study was permitted by the Ethics Committee of Nanjing Drum Tower Hospital.

### Popliteal LN metastasis model

4-week-old female nude BALB/c mice were purchased from the Animal Core Facility of Nanjing Medical University (Nanjing, China). The mice were divided randomly into groups, and 5 × 10^6^ stably transfected T24 cells were inoculated into the footpads of the mice. The mice were sacrificed after four weeks, and the primary tumor and popliteal LNs were excised for further histological analysis.

### Next-generation sequencing

Next-generation sequencing was performed on 3 pairs of T24-LN5/T24-NC cells. The samples were prepared, and paired-end sequencing was performed on an Illumina HiSeq 4000 (LC bio, HangZhou, China). The upregulated circRNAs and mRNAs were filtered by log2 (fold change) > 1 and *p* < 0.05.

### RNA preparation and quantitative RT-PCR (qRT-PCR)

TRIzol reagent (Sigma, MO, USA) was used to extract RNAs. PARIS Kit (Life Technologies, MA, USA) was used to extract the nuclear and cytoplasmic fractions. mRNAs were reverse-transcribed with random primers, and miRNA with stem-loop primers using the Takara system (Takara, Japan). qRT-PCR was performed using ChamQ SYBR qPCR Master Mix (Vazyme, Nanjing, China). The 2^−∆∆Ct^ method was used to calculate the differences in gene expression. CircRNA and mRNA were normalized to GAPDH, and miRNA was normalized to U6. The primer sequences were shown in Table S1.

### Western blot

The proteins were extracted in RIPA buffer (Beyotime, Shanghai, China) containing 1% phosphatase and protease inhibitors. The concentration was detected using BCA protein assay kit (Thermo Scientific, IL, USA). The antibodies used included primary antibodies against CXCL5 (ab126763, Abcam, Cambridge, UK) and GAPDH (#5174, Cell Signaling Technology, MA, USA).

### Plasmid construction and oligonucleotide transfection

To construct circDHTKD1 overexpression plasmids, circDHTKD1 cDNA was cloned into the pLC5-ciR lentiviral vector by Geneseed (Guangzhou, China). siRNAs, miRNA mimics, and miRNA inhibitors were synthesized by GenePharma (Shanghai, China). Transfection was performed using Lipofectamine 3000 (Life Technologies, CA, USA).

### Wound healing assay

5637 or T24 cells were cultured in 6-well plates, and the cell monolayer was scratched with the fine end of a 200 μl pipette tip. Photographs were taken at 0 and 24 h to measure the area occupied by migratory cells.

### HLEC tube formation assay and Transwell assay

5 × 10^4^ HLECs were cultured in 96-well plates that were pre-coated with Matrigel (BD Biosciences, CA, USA). Then culture medium preconditioned with BCa cells was added into the plate, and incubated for 5 h. The lymphatic tubes were imaged with a microscope, and the length of tubes was measured and quantified.

For the migration assay, HLECs were cultured with conditioned medium of 5637 or T24 cells for 24 h. Then, 1 × 10^5^ HLECs in serum-free culture medium were inoculated in the upper chamber of Transwell chambers (Corning, NY, USA), and 500 μl culture media with FBS was applied to the bottom chamber. For the invasion assay, 1 × 10^5^ tumor cells were inoculated in the upper chamber pre-coated with Matrigel. After incubation for approximately 24 h, the cells on the lower surface of the filter were fixed and stained with crystal violet. Images were taken from five random fields and the cells were calculated.

### CCK-8 and colony formation assay

For CCK-8 assay, tumor cells were seeded in 96-well plates (5 × 10^3^ cells per well). At the indicated time points, the cells were treated with 10 μl of CCK-8 solution (Dojindo, Japan), and the cell viability was detected at OD450 on a microplate reader.

For colony formation assay, tumor cells were seeded into 12-well plates (300 cells per well) and incubated for 2 weeks. The cells were fixed in 4% paraformaldehyde and stained with 0.1% crystal violet. The visible colonies were counted and photographed.

### RNA Pull-down assay

The biotinylated circDHTKD1 probe or oligo probe (Tsingke, Wuhan, China) was incubated with streptavidin magnetic beads (Life Technologies, USA) at 25 °C for 2 h. The lysates of 1 × 10^7^ cells were incubated with prepared beads at 4 °C overnight. The RNA complexes were harvested using TRIzol reagent.

For biotinylated miRNA capture, BCa cells were transfected with biotinylated miRNA mimics or control (Tsingke, Wuhan, China). The cell lysates were incubated with streptavidin magnetic beads for 3 hours. TRIzol was used to harvest the RNA complex.

### Fluorescence in situ hybridization (FISH)

Cy3-labelled circDHTKD1 probes and Fam-labelled miR-149-5p probes were synthesized by GenePharma (Shanghai, China). Fluorescent In Situ Hybridization Kit (GenePharma, Shanghai, China) was used to conduct the assays. All photographs were taken on Lei TCS SP8 Laser Scanning Confocal Microscope (Leica Microsystems, Mannheim, Germany).

### Luciferase activity assay

The CXCL5 3′-UTR containing the predicted miR-149-5p binding site was inserted into the p-MIR-reporter plasmid. Wild-type or mutant plasmids were co-transfected with miR-149-5p mimics or mimic NC into HEK293T cells. After 48 hours, the luciferase activities were measured using a luciferase assay kit (Promega, WI, USA), and relative Renilla luciferase activity was normalized.

### Enzyme-linked immunosorbent assay (ELISA)

The concentration of CXCL5 in cell culture medium was measured using an ELISA kit (mlbio, Shanghai, China).

### Immunohistochemistry (IHC)

The IHC assays were performed as previously described [14]. The tissue sections were incubated with primary antibodies against LYVE-1 (Novus Biologicals, CO, USA) or Ly-6G (BD Pharmingen, NJ, USA). The degree of IHC in each section was reviewed and scored by two independent pathologists.

### Isolation of human peripheral blood neutrophils

Polymorphprep (Axis-Shield PoC AS, Oslo, Norway) was used to isolate neutrophils from the peripheral blood of healthy donors. The RBCs were lysed using a hypotonic lysis procedure. Neutrophils were cultured in 1640 medium.

### Neutrophil chemotaxis assay

1 × 10^6^ neutrophils were inoculated into the upper chamber (4 µm pore) in serum-free medium. Conditioned medium was applied to the bottom chamber. After incubation for 2 h, the migrated neutrophils were collected and calculated.

### Statistical analysis

The results are expressed as mean ± standard deviation (SD). Student’s *t* test or Chi-square test were used to analyze the differences between two groups. Pearson correlation coefficient was used to analyze the correlation. Survival was evaluated by Kaplan–Meier analysis. A *p* value < 0.05 was considered as statistical significance.

## Supplementary information

Supplementary figure and table legends

Table S1

Figure S1

Figure S2

Figure S3

Figure S4

Figure S5

Original data

## Data Availability

The datasets used and analyzed during the current study are available from the corresponding author on reasonable request.

## References

[1] Antoni S, Ferlay J, Soerjomataram I, Znaor A, Jemal A, Bray F (2017). Bladder Cancer Incidence and Mortality: A Global Overview and Recent Trends[J]. Eur Urol.

[2] van Rhijn BW, Burger M, Lotan Y, Solsona E, Stief CG, Sylvester RJ (2009). Recurrence and progression of disease in non-muscle-invasive bladder cancer: from epidemiology to treatment strategy[J]. Eur Urol.

[3] Dy GW, Gore JL, Forouzanfar MH, Naghavi M, Fitzmaurice C (2017). Global Burden of Urologic Cancers, 1990-2013[J]. Eur Urol.

[4] Soloway MS (2013). Bladder cancer: Lack of progress in bladder cancer-what are the obstacles?[J]. Nat Rev Urol.

[5] Xie R, Chen X, Cheng L, Huang M, Zhou Q, Zhang J (2021). NONO Inhibits Lymphatic Metastasis of Bladder Cancer via Alternative Splicing of SETMAR[J]. Mol Ther.

[6] Karaman S, Detmar M (2014). Mechanisms of lymphatic metastasis[J]. J Clin Invest.

[7] Stacker SA, Williams SP, Karnezis T, Shayan R, Fox SB, Achen MG (2014). Lymphangiogenesis and lymphatic vessel remodelling in cancer[J]. Nat Rev Cancer.

[8] Skobe M, Hawighorst T, Jackson DG, Prevo R, Janes L, Velasco P (2001). Induction of tumor lymphangiogenesis by VEGF-C promotes breast cancer metastasis[J]. Nat Med.

[9] Wang Y, Mo Y, Gong Z, Yang X, Yang M, Zhang S (2017). Circular RNAs in human cancer[J]. Mol Cancer.

[10] Rybak-Wolf A, Stottmeister C, Glazar P, Jens M, Pino N, Giusti S (2015). Circular RNAs in the Mammalian Brain Are Highly Abundant, Conserved, and Dynamically Expressed[J]. Mol Cell.

[11] Salzman J, Chen RE, Olsen MN, Wang PL, Brown PO (2013). Cell-type specific features of circular RNA expression[J]. PLoS Genet.

[12] Memczak S, Jens M, Elefsinioti A, Torti F, Krueger J, Rybak A (2013). Circular RNAs are a large class of animal RNAs with regulatory potency[J]. Nature.

[13] Kristensen LS, Hansen TB, Veno MT, Kjems J (2018). Circular RNAs in cancer: opportunities and challenges in the field[J]. Oncogene.

[14] Lu Q, Liu T, Feng H, Yang R, Zhao X, Chen W (2019). Circular RNA circSLC8A1 acts as a sponge of miR-130b/miR-494 in suppressing bladder cancer progression via regulating PTEN[J]. Mol Cancer.

[15] Liu T, Lu Q, Liu J, Xie S, Feng B, Zhu W (2020). Circular RNA FAM114A2 suppresses progression of bladder cancer via regulating NP63 by sponging miR-762[J]. Cell Death Dis.

[16] Hansen TB, Jensen TI, Clausen BH, Bramsen JB, Finsen B, Damgaard CK (2013). Natural RNA circles function as efficient microRNA sponges[J]. Nature.

[17] Sumazin P, Yang X, Chiu HS, Chung WJ, Iyer A, Llobet-Navas D (2011). An extensive microRNA-mediated network of RNA-RNA interactions regulates established oncogenic pathways in glioblastoma[J]. Cell.

[18] Zhu J, Luo Y, Zhao Y, Kong Y, Zheng H, Li Y (2021). circEHBP1 promotes lymphangiogenesis and lymphatic metastasis of bladder cancer via miR-130a-3p/TGFbetaR1/VEGF-D signaling[J]. Mol Ther.

[19] Yang C, Mou Z, Zhang Z, Wu S, Zhou Q, Chen Y (2021). Circular RNA RBPMS inhibits bladder cancer progression via miR-330-3p/RAI2 regulation[J]. Mol Ther Nucleic Acids.

[20] Zheng Q, Bao C, Guo W, Li S, Chen J, Chen B (2016). Circular RNA profiling reveals an abundant circHIPK3 that regulates cell growth by sponging multiple miRNAs[J]. Nat Commun.

[21] Soler-Cardona A, Forsthuber A, Lipp K, Ebersberger S, Heinz M, Schossleitner K (2018). CXCL5 Facilitates Melanoma Cell-Neutrophil Interaction and Lymph Node Metastasis[J]. J Invest Dermatol.

[22] Suzuki K, Morita T, Tokue A (2005). Vascular endothelial growth factor-C (VEGF-C) expression predicts lymph node metastasis of transitional cell carcinoma of the bladder[J]. Int J Urol.

[23] Cao R, Bjorndahl MA, Religa P, Clasper S, Garvin S, Galter D (2004). PDGF-BB induces intratumoral lymphangiogenesis and promotes lymphatic metastasis[J]. Cancer Cell.

[24] Platonova N, Miquel G, Regenfuss B, Taouji S, Cursiefen C, Chevet E (2013). Evidence for the interaction of fibroblast growth factor-2 with the lymphatic endothelial cell marker LYVE-1[J]. Blood.

[25] Cao R, Bjorndahl MA, Gallego MI, Chen S, Religa P, Hansen AJ (2006). Hepatocyte growth factor is a lymphangiogenic factor with an indirect mechanism of action[J]. Blood.

[26] Pold M, Zhu LX, Sharma S, Burdick MD, Lin Y, Lee PP (2004). Cyclooxygenase-2-dependent expression of angiogenic CXC chemokines ENA-78/CXC Ligand (CXCL) 5 and interleukin-8/CXCL8 in human non-small cell lung cancer[J]. Cancer Res.

[27] Gao Y, Guan Z, Chen J, Xie H, Yang Z, Fan J (2015). CXCL5/CXCR2 axis promotes bladder cancer cell migration and invasion by activating PI3K/AKT-induced upregulation of MMP2/MMP9[J]. Int J Oncol.

[28] Lee SJ, Kim JE, Kim ST, Lee J, Park SH, Park JO (2018). The Correlation Between Serum Chemokines and Clinical Outcome in Patients with Advanced Biliary Tract Cancer[J]. Transl Oncol.

[29] Chen C, Xu ZQ, Zong YP, Ou BC, Shen XH, Feng H (2019). CXCL5 induces tumor angiogenesis via enhancing the expression of FOXD1 mediated by the AKT/NF-kappaB pathway in colorectal cancer[J]. Cell Death Dis.

[30] Miyazaki H, Patel V, Wang H, Ensley JF, Gutkind JS, Yeudall WA (2006). Growth factor-sensitive molecular targets identified in primary and metastatic head and neck squamous cell carcinoma using microarray analysis[J]. Oral Oncol.

[31] Mollaoglu G, Jones A, Wait SJ, Mukhopadhyay A, Jeong S, Arya R (2018). The Lineage-Defining Transcription Factors SOX2 and NKX2-1 Determine Lung Cancer Cell Fate and Shape the Tumor Immune Microenvironment[J]. Immunity.

[32] Lin Y, Cheng L, Liu Y, Wang Y, Wang Q, Wang HL (2021). Intestinal epithelium-derived BATF3 promotes colitis-associated colon cancer through facilitating CXCL5-mediated neutrophils recruitment[J]. Mucosal Immunol.

[33] Mao Z, Zhang J, Shi Y, Li W, Shi H, Ji R (2020). CXCL5 promotes gastric cancer metastasis by inducing epithelial-mesenchymal transition and activating neutrophils[J]. Oncogenesis.

[34] Chen LL (2016). The biogenesis and emerging roles of circular RNAs[J]. Nat Rev Mol Cell Biol.

[35] Hansen TB, Kjems J, Damgaard CK (2013). Circular RNA and miR-7 in cancer[J]. Cancer Res.

