# Supplementary material for: circDHTKD1 promotes lymphatic metastasis of bladder cancer by upregulating CXCL5
Source: Cell Death Discov. 2022 May 3;8:243. doi: 10.1038/s41420-022-01037-x (PMC9065127; doi:10.1038/s41420-022-01037-x)
Supplement: Supplementary file 1 — Supplementary figure and table legends [file 41420_2022_1037_MOESM1_ESM.docx]

**Supplementary figure and table legends**

**Table S1** Primers and RNA sequences used in this study.

**Fig. S1 T24-LN5 cells have higher LN-metastatic capacity than T24-NC. A** Representative images of excised popliteal LNs and the measurement of the LN volume.

**Fig. S2 circDHTKD1 promotes the proliferation of BCa cells. A** and **B** CCK-8 assay showing the proliferation capability after circDHTKD1 overexpression in BCa cells. **C** and **D** CCK-8 assay showing the proliferation capability after circDHTKD1 knockdown in BCa cells. **E** and **F** Colony formation assay showing the proliferation capability after circDHTKD1 overexpression in BCa cells. **G** and **H** Colony formation assay showing the proliferation capability after circDHTKD1 knockdown in BCa cells. **I** The measurement of the primary footpad tumor volume after circDHTKD1 overexpression.

**Fig. S3 Co-expressed mRNAs of circDHTKD1. A** Detailed information of three co-expressed mRNAs, ALOX5AP, FAM133A, and CXCL5. **B** and **C** The expressions of three co-expressed mRNAs were detected after circDHTKD1 overexpression in BCa cells by qRT-PCR.

**Fig. S4 circDHTKD1** **facilitates lymphangiogenesis by targeting CXCL5. A** qRT-PCR confirmed the CXCL5 expression in BCa tissues and matched normal tissues. **B** Analysis of CXCL5 expression in BCa tissues according to LN status. **C** Tube formation and Transwell migration of HLEC cultured with conditioned medium from indicated cells. **D** Immunofluorescence confirmed the expression of CXCR2 in HLEC.

**Fig. S5 Overexpression and knockdown of miR-149-5p in BCa cells. A** qRT-PCR was used to assess the expression of miR-149-5p in BCa cells after overexpression. **B** qRT-PCR was used to assess the expression of miR-149-5p in BCa cells after knockdown.
