# Supplementary material for: circDHTKD1 promotes lymphatic metastasis of bladder cancer by upregulating CXCL5
Source: Cell Death Discov. 2022 May 3;8:243. doi: 10.1038/s41420-022-01037-x (PMC9065127; doi:10.1038/s41420-022-01037-x)
Supplement: Supplementary file 2 — Table S1 [file 41420_2022_1037_MOESM2_ESM.docx]

**Table S1** Primers and RNA sequences used in this study.

| **List of oligonucleotide sequences** | **5'--> 3'** |
| --- | --- |
| **Primers for PCR** |  |
| circDHTKD1 Forward | GGTCCGTGGTCGTTTGTTTC |
| circDHTKD1 Reverse | ATTCTCCAGCAGGGCTTGTC |
| linear-DHTKD1 Forward | AGCATGGTCATAAAGCTGCC |
| linear-DHTKD1 Reverse | TGCACCAGGGCTTGGATTTC |
| GAPDH Forward | CAATGACCCCTTCATTGACC |
| GAPDH Reverse | TTGATTTTGGAGGGATCTCG |
| divergent-GAPDH Forward | GAAGGTGAAGGTCGAGTC |
| divergent-GAPDH Reverse | GAAGATGGTGATGGGATTTC |
| CXCL5 Forward | GACGGTGGAAACAAGGAAAA |
| CXCL5 Reverse | GCTTAAGCGGCAAACATAGG |
| hsa-miR-24-3p RT | CTCAACTGGTGTCGTGGAGTCGGCAATTCAGTTGAGCTGTTCCT |
| hsa-miR-24-3p Forward | ACACTCCAGCTGGGTGGCTCAGTTCAGCAG |
| hsa-miR-149-5p RT | CTCAACTGGTGTCGTGGAGTCGGCAATTCAGTTGAGGGGAGTGA |
| hsa-miR-149-5p Forward | ACACTCCAGCTGGGTCTGGCTCCGTGTCTT |
| hsa-miR-199a-5p RT | CTCAACTGGTGTCGTGGAGTCGGCAATTCAGTTGAGGAACAGGT |
| hsa-miR-199a-5p Forward | ACACTCCAGCTGGGCCCAGTGTTCAGACTA |
| hsa-miR-345-5p RT | CTCAACTGGTGTCGTGGAGTCGGCAATTCAGTTGAGGAGCCCTG |
| hsa-miR-345-5p Forward | ACACTCCAGCTGGGGCTGACTCCTAGTCCA |
| hsa-miR-377-3p RT | CTCAACTGGTGTCGTGGAGTCGGCAATTCAGTTGAGACAAAAGT |
| hsa-miR-377-3p Forward | ACACTCCAGCTGGGATCACACAAAGGCAAC |
| hsa-miR-409-3p RT | CTCAACTGGTGTCGTGGAGTCGGCAATTCAGTTGAGAGGGGTTC |
| hsa-miR-409-3p Forward | ACACTCCAGCTGGGGAATGTTGCTCGGTGA |
| hsa-miR-432-5p RT | CTCAACTGGTGTCGTGGAGTCGGCAATTCAGTTGAGCCACCCAA |
| hsa-miR-432-5p Forward | ACACTCCAGCTGGGTCTTGGAGTAGGTCAT |
| hsa-miR-495-3p RT | CTCAACTGGTGTCGTGGAGTCGGCAATTCAGTTGAGAAGAAGTG |
| hsa-miR-495-3p Forward | ACACTCCAGCTGGGAAACAAACATGGTGCA |
| U6 RT | CTCAACTGGTGTCGTGGAGTCGGCAATTCAGTTGAGAACGCTTC |
| U6 Forward | ACACTCCAGCTGGGACGCAAATTCGTGAAG |
| unified reverse primer | TGGTGTCGTGGAGTCGGCAA |
| **siRNAs and shRNAs** |  |
| si NC sense | UUCUCCGAACGUGUCACGUTT |
| si NC antisense | ACGUGACACGUUCGGAGAATT |
| si circDHTKD1-1 sense | AGCUGGCCUGCAAGUUGAUTT |
| si circDHTKD1-1 antisense | AUCAACUUGCAGGCCAGCUTT |
| si circDHTKD1-2 sense | GGCCUGCAAGUUGAUCAUGTT |
| si circDHTKD1-2 antisense | CAUGAUCAACUUGCAGGCCTT |
| sh CXCL5 | GATCAGTAATCTGCAAGTGTT |
| **FISH probes** |  |
| circDHTKD1 | GCCATGATCAACTTGCAGGCCAGCT |
| miR-149-5p | GGGAGTGAAGACACGGAGCCAGA |
| **Biotinylated probes** |  |
| biotin-NC | AGACCGGCCGAATTAGTCCTCGTCA |
| biotin-circDHTKD1-1 | GCCATGATCAACTTGCAGGCCAGCT |
| biotin-circDHTKD1-2 | GGCAAGGCCATGATCAACTTGCAGG |
| biotin-mimic NC sense | UUCUCCGAACGUGUCACGUTT |
| biotin-mimic NC antisense | ACGUGACACGUUCGGAGAATT |
| biotin-miR-149-5p mimic sense | UCUGGCUCCGUGUCUUCACUCCC |
| biotin-miR-149-5p mimic antisense | GAGUGAAGACACGGAGCCAGAUU |
| **miRNA mimics and inhibitors** |  |
| mimic NC sense | UUCUCCGAACGUGUCACGUTT |
| mimic NC antisense | ACGUGACACGUUCGGAGAATT |
| miR-149-5p mimic sense | UCUGGCUCCGUGUCUUCACUCCC |
| miR-149-5p mimic antisense | GAGUGAAGACACGGAGCCAGAUU |
| inhibitor NC | UCUACUCUUUCUAGGAGGUUGUGA |
| miR-149-5p inhibitor | GGGAGUGAAGACACGGAGCCAGA |
|  |  |
|  |  |
|  |  |
|  |  |
|  |  |
|  |  |
|  |  |
|  |  |
|  |  |
