# Supplementary figures and images for: circDHTKD1 promotes lymphatic metastasis of bladder cancer by upregulating CXCL5
Source: Cell Death Discov. 2022 May 3;8:243. doi: 10.1038/s41420-022-01037-x (PMC9065127; doi:10.1038/s41420-022-01037-x)

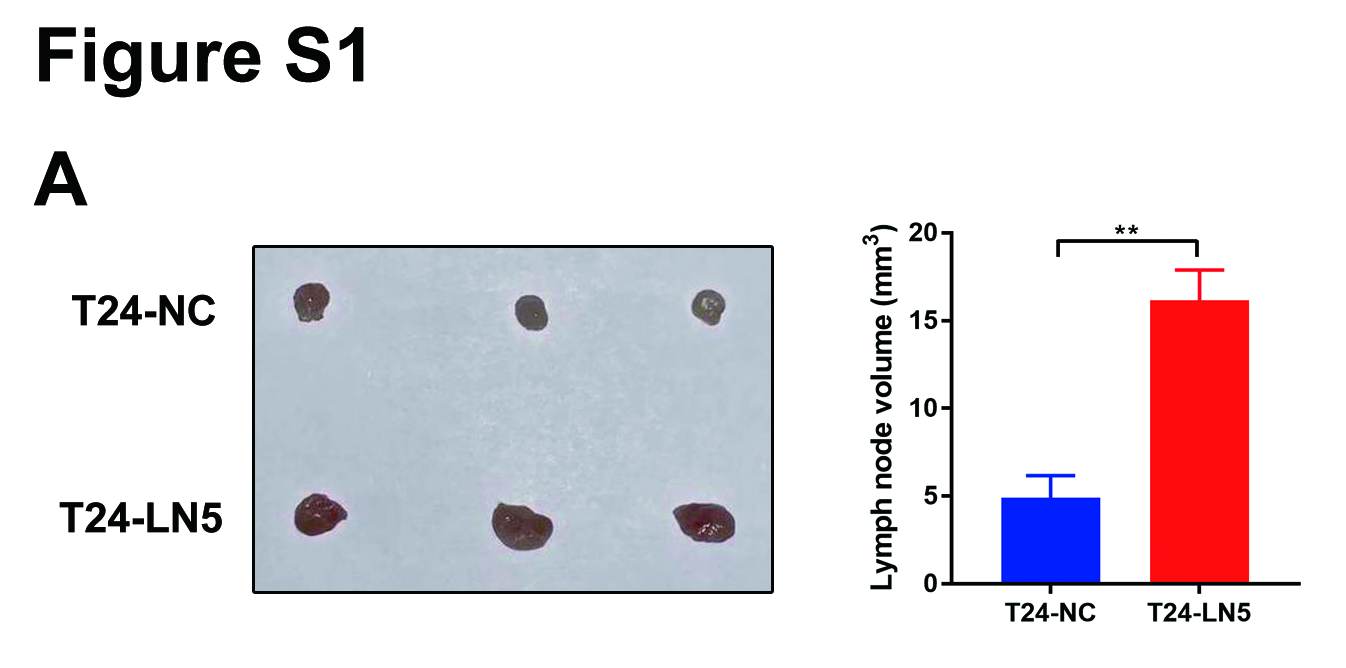

Supplement: Supplementary file 3 — Figure S1 [file 41420_2022_1037_MOESM3_ESM.tif]

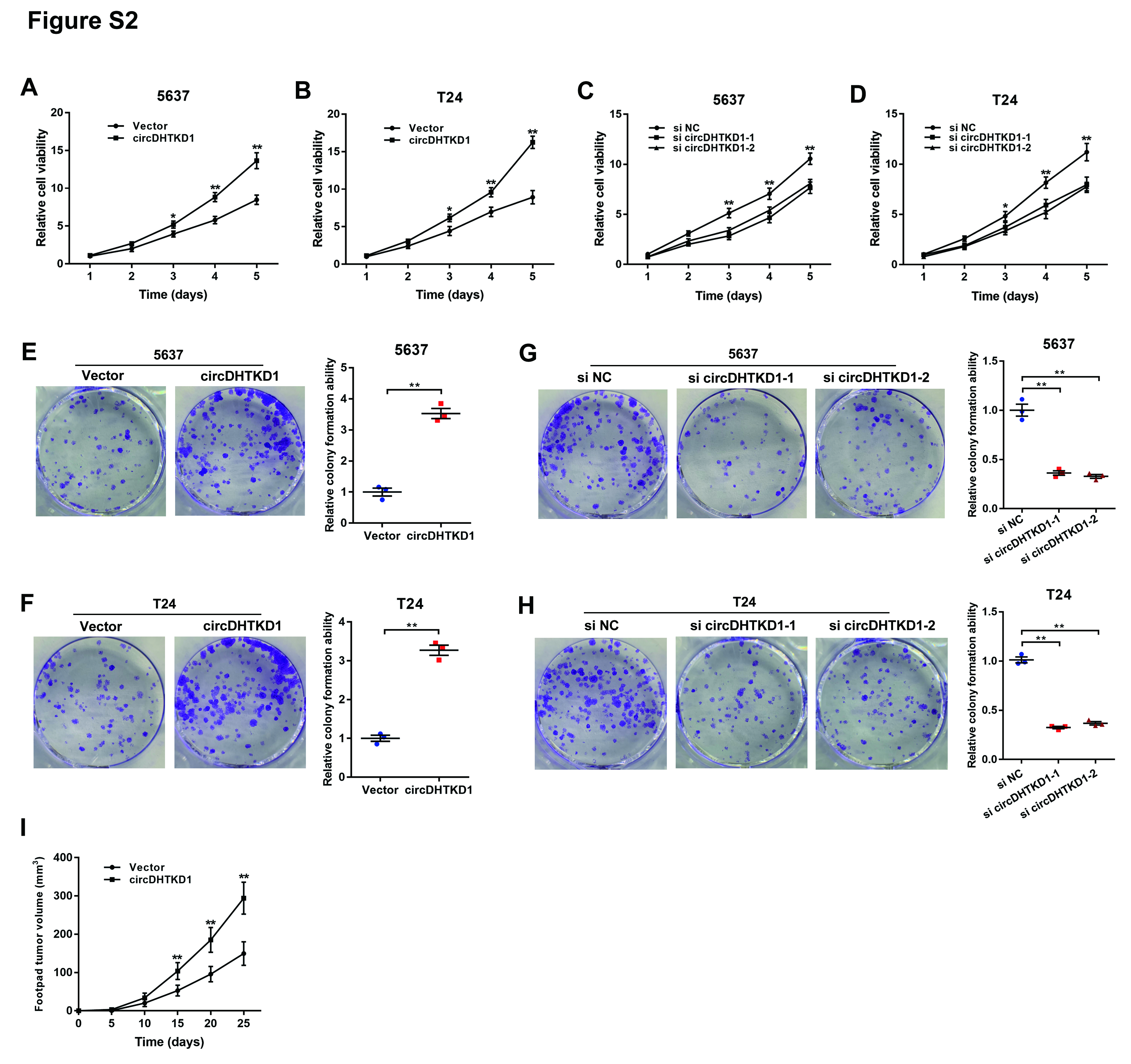

Supplement: Supplementary file 4 — Figure S2 [file 41420_2022_1037_MOESM4_ESM.tif]

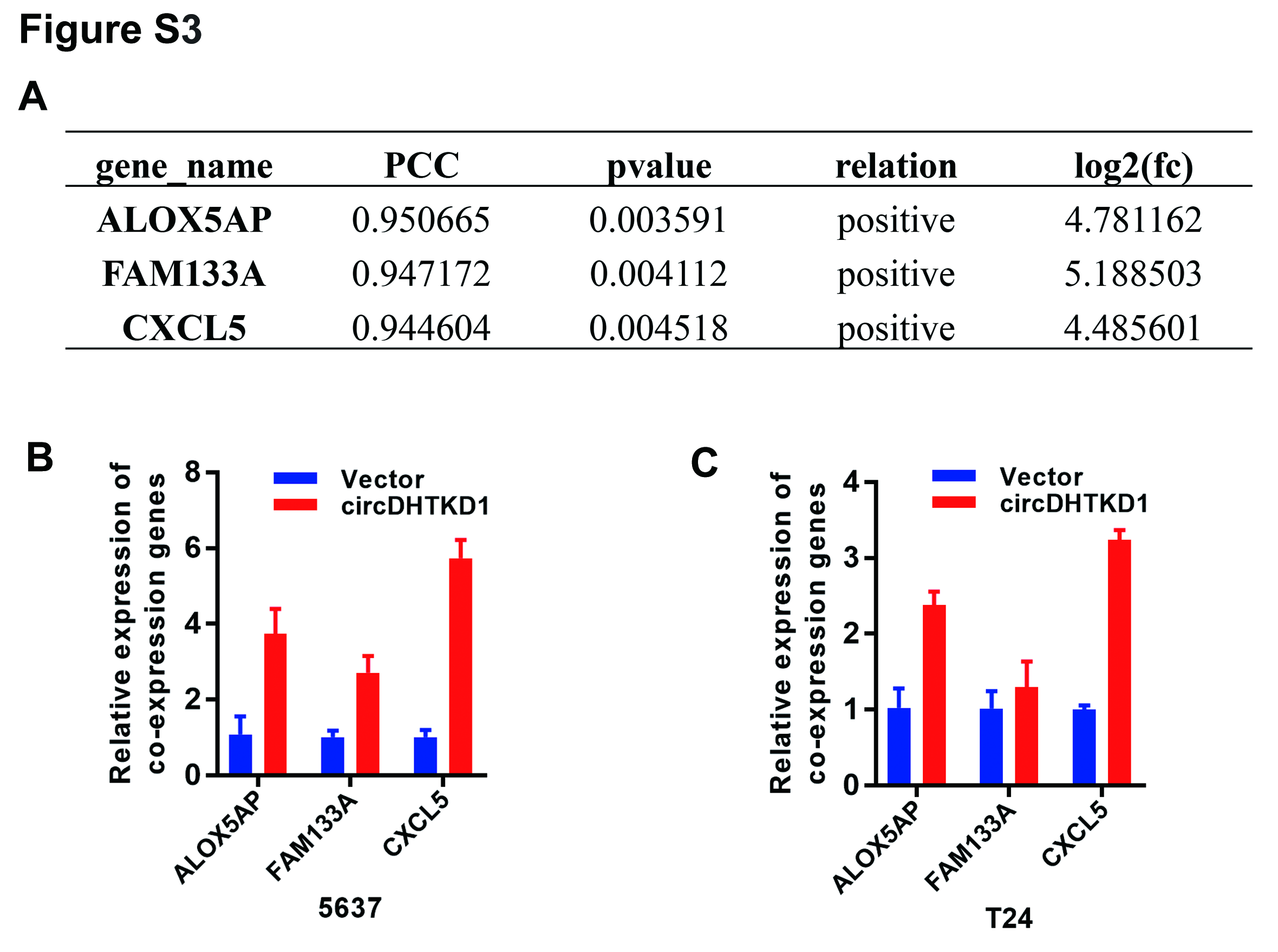

Supplement: Supplementary file 5 — Figure S3 [file 41420_2022_1037_MOESM5_ESM.tif]

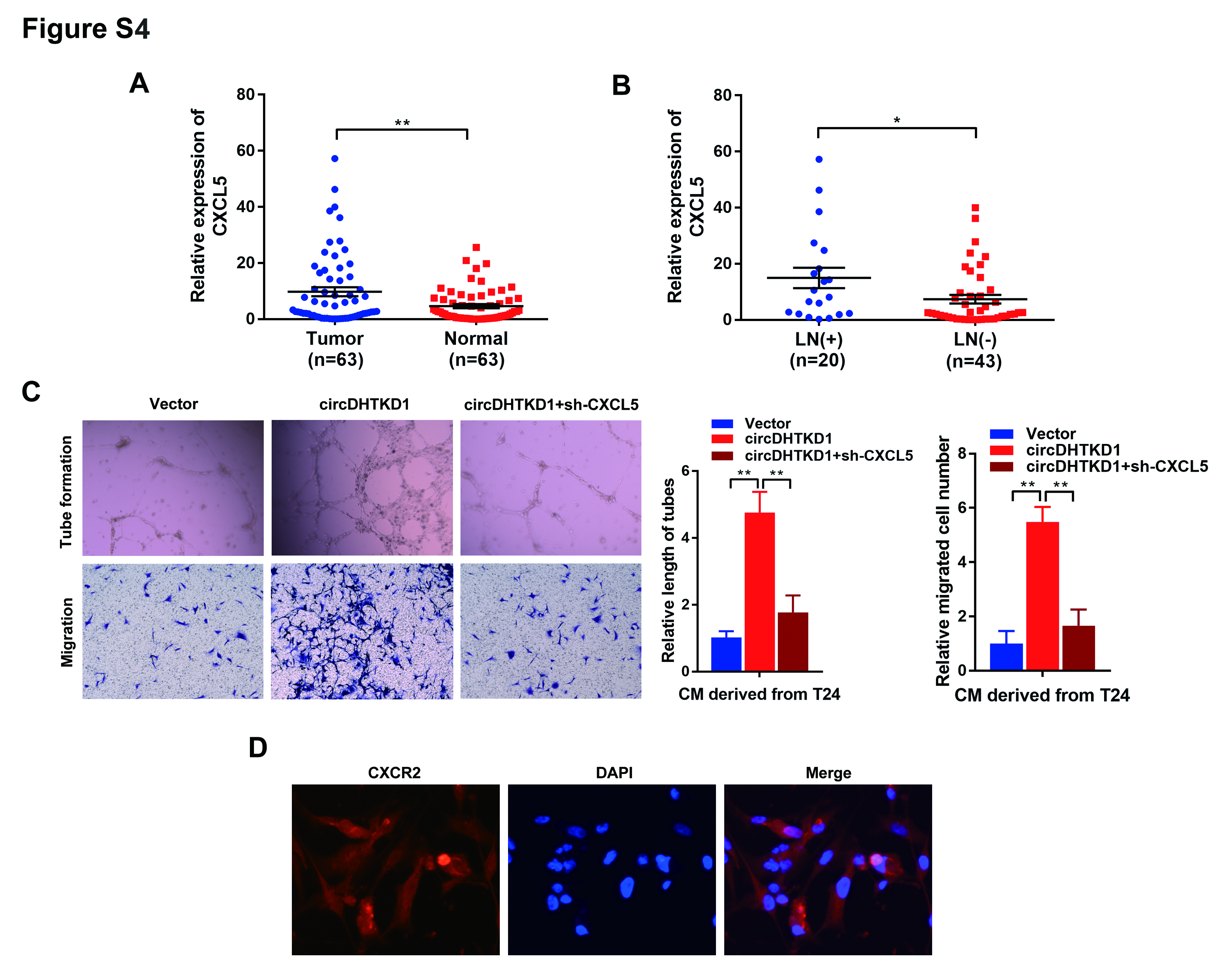

Supplement: Supplementary file 6 — Figure S4 [file 41420_2022_1037_MOESM6_ESM.tif]

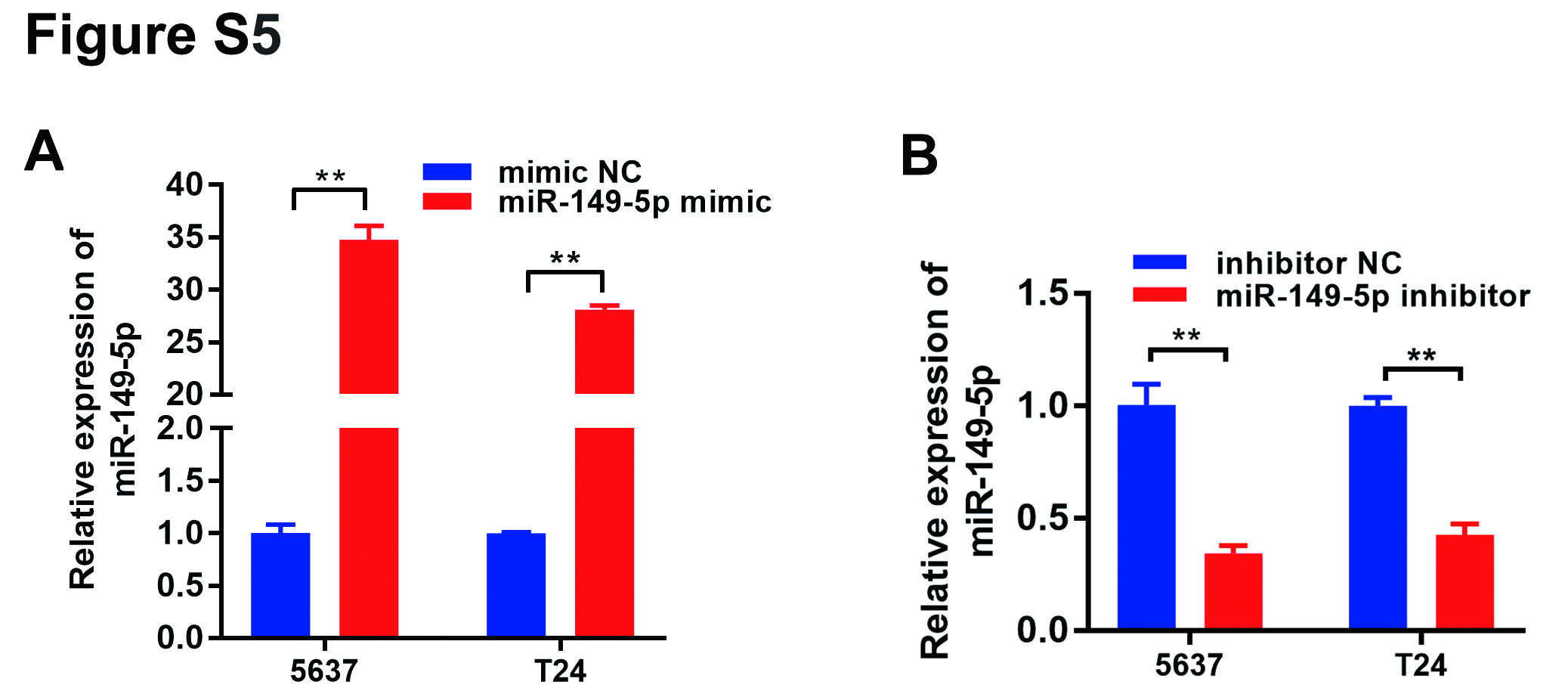

Supplement: Supplementary file 7 — Figure S5 [file 41420_2022_1037_MOESM7_ESM.tif]

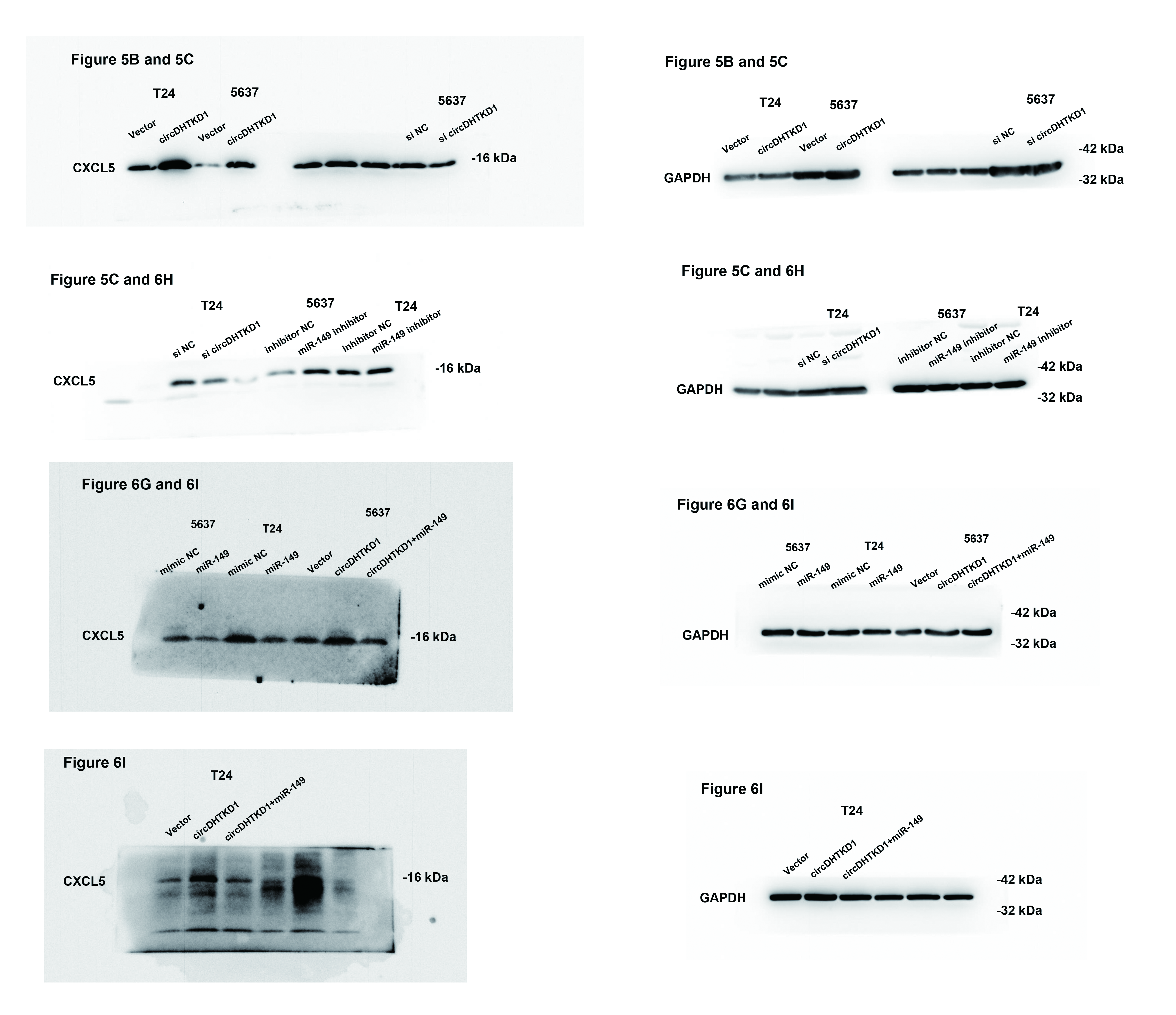

Supplement: Supplementary file 8 — Original data [file 41420_2022_1037_MOESM8_ESM.tif]
